# Preoperative calculation of risk for prolonged intensive care unit stay following coronary artery bypass grafting

**DOI:** 10.1186/1749-8090-1-14

**Published:** 2006-05-31

**Authors:** Sanjay V Ghotkar, Antony D Grayson, Brian M Fabri, Walid C Dihmis, D Mark Pullan

**Affiliations:** 1 Department of Cardiothoracic Surgery, The Cardiothoracic Centre, Liverpool, UK; 2 Clinical Governance Department, The Cardiothoracic Centre, Liverpool, UK

## Abstract

**Objective:**

Patients who have prolonged stay in intensive care unit (ICU) are associated with adverse outcomes. Such patients have cost implications and can lead to shortage of ICU beds. We aimed to develop a preoperative risk prediction tool for prolonged ICU stay following coronary artery surgery (CABG).

**Methods:**

5,186 patients who underwent CABG between 1st April 1997 and 31st March 2002 were analysed in a development dataset. Logistic regression was used with forward stepwise technique to identify preoperative risk factors for prolonged ICU stay; defined as patients staying longer than 3 days on ICU. Variables examined included presentation history, co-morbidities, catheter and demographic details. The use of cardiopulmonary bypass (CPB) was also recorded. The prediction tool was tested on validation dataset (1197 CABG patients between 1^st ^April 2003 and 31^st ^March 2004). The area under the receiver operating characteristic (ROC) curve was calculated to assess the performance of the prediction tool.

**Results:**

475(9.2%) patients had a prolonged ICU stay in the development dataset. Variables identified as risk factors for a prolonged ICU stay included renal dysfunction, unstable angina, poor ejection fraction, peripheral vascular disease, obesity, increasing age, smoking, diabetes, priority, hypercholesterolaemia, hypertension, and use of CPB. In the validation dataset, 8.1% patients had a prolonged ICU stay compared to 8.7% expected. The ROC curve for the development and validation datasets was 0.72 and 0.74 respectively.

**Conclusion:**

A prediction tool has been developed which is reliable and valid. The tool is being piloted at our institution to aid resource management.

## 1. Introduction

Coronary artery bypass graft (CABG) surgery mortality rates have declined significantly over the last decade, despite an increase in older, sicker, and higher-risk patients being treated [1]. However, the incidence of post-operative morbidity has been increasing, which can lead to prolonged lengths of stay in hospital for patients, in particular the intensive care unit (ICU).

Intensive care not only requires the use of sophisticated equipment, but also highly skilled and dedicated nursing and medical staff. As such, the ICU takes up a significant proportion of the total cost associated with a patients overall hospital stay, and therefore, patients with prolonged ICU stays can have serious cost implications. Furthermore, patients with prolonged ICU stay can also lead to a shortage of ICU beds and result in operations being cancelled.

Several papers have attempted to identify preoperative risk factors associated with prolonged ICU stay, but they have been on relatively small numbers [2-5]. Jansen and associates recently published a logistic regression equation to predict the risk of patients staying in the ICU for more than 3 days, however, this was limited by the fact that it was only based on a total of 104 outcomes [5].

In a large cohort, we aimed to identify patient and disease characteristics associated with prolonged ICU stay and to develop and validate a risk prediction model to estimate the risk of prolonged intensive care.

## 2. Methods

### 2.1 Patient population and data

We performed a retrospective study on a total of 5,186 consecutive patients undergoing CABG surgery between 1st April 1997 and 31st March 2002 at the Cardiothoracic Centre-Liverpool. Patients undergoing CABG that was combined with a heart valve repair or replacement, resection of a ventricular aneurysm or other surgical procedures were not included. Approval for the study was given by the hospitals Cardiothoracic Surgery Division and by the Research and Development Department.

Data was collected prospectively during the patient's admission as part of routine clinical practice on the following variables: age, sex, body mass index (BMI), urgency of operation, prior cardiac surgery or percutaneous coronary interventions, New York Heart Association functional class, Canadian Cardiovascular Society angina class, the extent of coronary disease, and left ventricular ejection fraction. History of myocardial infarction, smoking, diabetes, hypercholesterolemia, hypertension, peripheral vascular disease, cerebrovascular disease, respiratory disease, renal dysfunction, gastric ulcer, and gastrointestinal surgery were also noted. The use of cardiopulmonary bypass (CPB) was also recorded. Definitions and data collection methods have been previously published [6] and are available at www.nwheartaudit.nhs.uk.

### 2.2 Prolonged ICU stay

Criteria for discharge from the ICU included cardiovascular stability, minimal or no respiratory assistance, evidence of adequate renal function with normal serum electrolyte levels, and evidence of adequate neuropsychological function. Days spent in the ICU were counted by patient census at midnight each day. Patients who stayed in the ICU for more than 3 consecutive days on the initial admission were classified as having a prolonged ICU stay, while patients staying 3 days or less were classified as having a normal ICU stay.

### 2.3 Statistical analysis

Continuous data are shown as median values with 25th and 75th percentiles. Categorical variables are shown as a percentage and comparisons were made with Chi-square tests as appropriate. Standard statistical tests were used to calculate odds ratios and 95% confidence intervals. A multivariate logistic regression analysis was undertaken, using the forward stepwise technique, to identify independent risk factors for prolonged ICU stay [7]. Candidate variables were entered into the model with a p-value less than 0.1. The area under the receiver operating characteristic (ROC) curve and the Hosmer-Lemeshow goodness-of-fit statistic were calculated to assess the performance and calibration of the model, respectively [7,8]. The multivariate risk prediction model was compared against existing risk stratification tools: Parsonnet score [9], additive EuroSCORE [10], and logistic EuroSCORE [11].

A simplified clinical risk assessment tool was developed from the multivariate risk prediction model and was scored by rounding the adjusted odds ratio for each variable to the nearest 0.5. These weights were then summed. The relationship between this clinical risk score and the probability calculated from the risk prediction model was read from a graph. This clinical risk assessment tool therefore approximates the risk that would have been calculated from the risk prediction model. The clinical risk assessment tool was split into low (bottom 45% of cohort), medium, high (top 10% of cohort) risk groups, which may prove useful in aiding resource management.

External validation of the model was carried out on 1,197 consecutive isolated CABG cases covering the time period 1st April 2003 to 31st March 2004. In all cases a p-value < 0.05 was considered significant. All statistical analysis was performed with SAS for Windows Version 8.2. Due to a number of patients, 30 in total, who died on post-operative day three or earlier, the data was re-analysed with these patients excluded to assess the effect that these patients might have on our conclusions. The independent risk factors identified originally remained unchanged and no significant differences occurred with respect to the weightings given to each variable.

## 3. Results

### 3.1 Outcomes and patient data

Of the 5,186 patients who underwent CABG, 475 (9.2%) had a prolonged ICU stay. The patient preoperative characteristics are reported in Table 1.

**Table 1:** Univariate association between preoperative characteristics and prolonged intensive care unit stay

	% of Patients	Prolonged ICU Stay (%)	Odds Ratio (95% CI)	P Value
Age (years)				
<70	75.4	7.9	Ref.	
70 – 74	16.6	12.4	1.6 (1.3 – 2.1)	<0.001
≥ 75	8.0	14.4	2.0 (1.5 – 2.6)	<0.001
			Trend	<0.001
Gender				
Male	80.5	9.1	Ref.	
Female	19.5	9.6	1.1 (0.8 – 1.3)	0.58
Body mass index (kg/m^2^)				
<30	72.8	8.4	Ref.	
30 – 34	22.1	10.4	1.3 (1.0 – 1.6)	0.035
≥ 30	5.1	15.0	1.9 (1.3 – 2.7)	<0.001
			Trend	<0.001
Angina class IV				
No	68.1	7.3	Ref.	
Yes	31.9	13.1	1.9 (1.6 – 2.3)	<0.001
NYHA class IV				
No	93.9	8.7	Ref.	
Yes	6.1	15.5	1.9 (1.4 – 2.6)	<0.001
Previous myocardial infarction				
No	53.5	8.0	Ref.	
Yes	46.5	10.5	1.4 (1.1 – 1.6)	0.001
Recent myocardial infarction				
No	94.7	8.8	Ref.	
Yes	5.3	14.8	1.8 (1.3 – 2.5)	<0.001
Current smoker				
No	84.9	8.5	Ref.	
Yes	15.1	12.8	1.6 (1.2 – 2.0)	<0.001
Hypercholesterolaemia				
No	22.1	8.0	Ref.	
Yes	77.9	9.5	1.2 (0.9 – 1.5)	0.13
Hypertension				
No	49.0	7.4	Ref.	
Yes	51.0	10.8	1.5 (1.2 – 1.8)	<0.001
Diabetes				
No	83.6	8.2	Ref.	
Yes	16.4	14.2	1.9 (1.5 – 2.3)	<0.001
Renal dysfunction				
No	97.8	8.5	Ref.	
Yes	2.2	39.8	7.1 (4.8 – 10.5)	<0.001
Cerebrovascular disease				
No	91.7	8.8	Ref.	
Yes	8.3	13.3	1.6 (1.2 – 2.1)	0.002
Peripheral vascular disease				
No	87.2	8.2	Ref.	
Yes	12.8	15.9	2.1 (1.7 – 2.7)	<0.001
Respiratory disease				
No	69.2	8.2	Ref.	
Yes	30.8	11.3	1.4 (1.2 – 1.7)	<0.001
Previous gastric ulcer				
No	88.4	9.0	Ref.	
Yes	11.6	10.3	1.2 (0.9 – 1.5)	0.28
Previous GI surgery				
No	88.3	8.9	Ref.	
Yes	11.7	11.2	1.3 (1.0 – 1.7)	0.061
Previous PCI				
No	94.1	9.2	Ref.	
Yes	5.9	9.2	1.0 (0.7 – 1.5)	0.97
Ejection fraction <30%				
No	91.0	8.2	Ref.	
Yes	9.0	18.7	2.6 (2.0 – 3.3)	<0.001
Triple-vessel disease				
No	18.2	6.5	Ref.	
Yes	81.8	9.7	1.6 (1.2 – 2.1)	0.002
Left main stenosis >50%				
No	81.9	9.2	Ref.	
Yes	18.1	9.2	1.0 (0.8 – 1.3)	>0.99
Prior Heart surgery				
No	97.3	9.1	Ref.	
Yes	2.7	12.9	1.5 (0.9 – 2.4)	0.12
Emergency procedure				
No	97.9	8.8	Ref.	
Yes	2.1	24.6	3.4 (2.1 – 5.2)	<0.001

ICU, Intensive Care Unit; CI, Confidence Intervals; NYHA, New York Heart Association; GI, Gastro-intestinal; PCI, Percutaneous Coronary Interventions.

### 3.2 Univariate association with prolonged ICU stay

Table 1 shows the univariate association with with prolonged ICU stay. Significant preoperative characteristics included age, body mass index, angina class, NYHA class, history of myocardial infarction, smoking, hypertension, diabetes, renal dysfunction, cerebrovascular disease, peripheral vascular disease, respiratory disease, ejection fraction, extent of disease, and urgency of operation. 4381 (84.5%) patients underwent CABG with CPB while the remaining 805 (15.5%) underwent CABG without CPB. In the patients with CPB used, 9.9% had a prolonged ICU stay compared to 5.2% in patients without CPB (p < 0.001).

### 3.3 Independent risk factors for prolonged ICU stay

**Figure 1:**
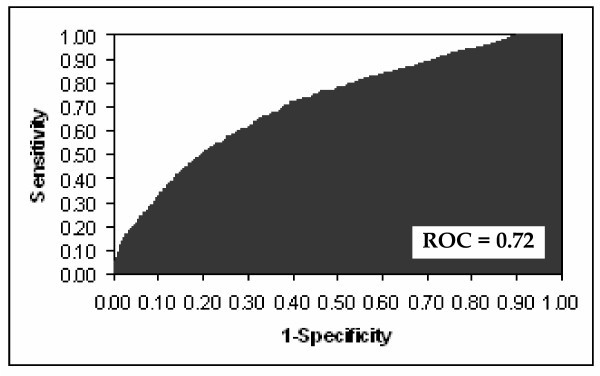
Receiver operator characteristic curve for multivariate prediction model.

**Figure 2:**
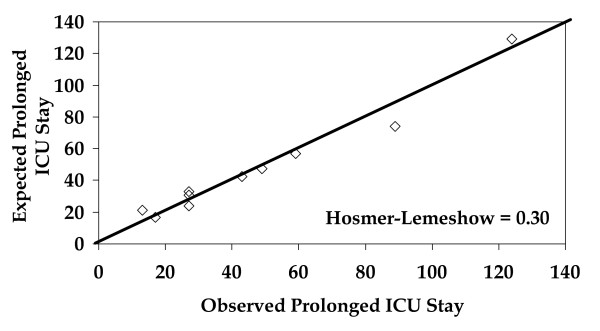
Hosmer-Lemeshow plot of observed number of patients with a prolonged intensive care unit stay (x-axis) versus predicted number of patients with a prolonged intensive care unit stay (y-axis) by decile of risk.

The independent risk factors for prolonged ICU stay, along with co-efficients, standard errors, odds ratios, confidence limits, and p-values, are shown in Table 2. The logistic regression equation for calculation of predicted risk of prolonged ICU stay is shown at the bottom of Table 2. The ROC curve for the multivariate prediction model was 0.72 (Figure 1). The predicted risks of individual patients were rank-ordered and divided into deciles. Within each group of estimated risk, the number of prolonged ICU stay predicted was compared with the number of observed prolonged ICU stay. The Hosmer-Lemeshow goodness-of-fit statistic across groups of risk was not statistically significant (Figure 2, p = 0.30), indicating little departure from a perfect fit.

**Table 2:** Independent risk factors for prolonged intensive care unit stay

	Co-efficient	SE	Adjusted OR (95% CI)	P Value
Renal dysfunction	1.6066	0.2162	4.99 (3.3 – 7.6)	<0.001
Angina class IV	0.4950	0.1048	1.64 (1.3 – 2.0)	<0.001
Ejection fraction <30%	0.7771	0.1397	2.17 (1.6 – 2.9)	<0.001
Peripheral vascular disease	0.4809	0.1288	1.62 (1.2 – 2.1)	<0.001
BMI ≥ 30 and <35 kg/m2	0.3338	0.1194	1.39 (1.1 – 1.8)	0.005
BMI ≥ 35 kg/m2	0.7436	0.1935	2.1 (1.4 – 3.1)	<0.001
Age ≥ 70 and <75 years	0.5313	0.1283	1.7 (1.3 – 2.2)	<0.001
Age ≥ 75 years	0.7972	0.1640	2.22 (1.6 – 3.1)	<0.001
Current smoker	0.5238	0.1291	1.69 (1.3 – 2.2)	<.0001
Diabetes	0.4381	0.1207	1.55 (1.2 – 2.0)	<0.001
Emergent procedure	0.7124	0.2510	2.04 (1.2 – 3.3)	0.004
Hypercholesterolaemia	0.3507	0.1282	1.42 (1.1 – 1.8)	0.006
Hypertension	0.2577	0.1039	1.29 (1.1 – 1.6)	0.013
Use of CPB	0.8904	0.1734	2.44 (1.7 – 3.4)	<0.001
Intercept	-4.4390			

Calculation of predicted risk using patient data and logistic regression co-efficients:

Calculate the odds of prolonged intensive care unit stay = exp (-4.4390 + [1.6066 × renal dysfunction] + [0.4950 × angina class IV] + [0.7771 × ejection fraction <30%] + [0.4809 × peripheral vascular disease] + [0.3338 × BMI ≥ 30 and <35 kg/m2] + [0.7436 × BMI ≥ 35 kg/m2] + [0.5313 × age ≥ 70 and < 75 years] + [0.7972 × age ≥ 75 years] + [0.5238 × current smoker] + [0.4381 × diabetes] + [0.7124 × emergent procedure] + [0.3507 × hypercholesterolaemia] + [0.2577 × hypertension] + [0.8904 × use of CPB]).

Predicted risk of prolonged intensive care unit stay as a percentage = [odds/(1 + odds)] × 100.

SE, Standard Error; OR, Odds Ratio; CI, Confidence Intervals; BMI, Body Mass Index; CPB, Cardiopulmonary Bypass.

### 3.4 Comparison with existing risk stratification tools

**Figure 3:**
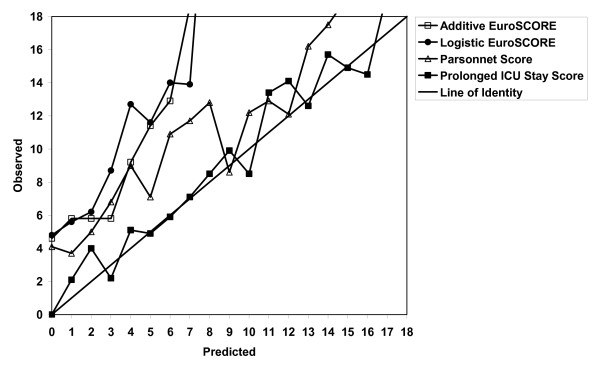
Comparison of multivariate prediction model for prolonged intensive care unit stay with existing risk stratification tools.

Figure 3 compares the logistic regression equation for prolonged ICU stay with three existing risk stratification tools. The logistic regression equation was a better predictor compared to the Parsonnet score (ROC curve = 0.65), additive EuroSCORE (ROC curve = 0.66), and the logistic EuroSCORE (ROC curve = 0.66).

### 3.5 Validation of the model

Applying the logistic regression equation to data for 1,197 consecutive CABG cases performed between 1st April 2003 and 31st March 2004 revealed a ROC curve of 0.74. The Hosmer- Lemeshow goodness-of-fit statistic across groups of risk was not statistically significant (p = 0.79), indicating little departure from a perfect fit. In this validation dataset the logistic regression model predicted 8.7% patients with prolonged ICU stay compared to 8.1% observed (p = 0.61).

### 3.6 Simplified scoring tool

**Figure 4:**
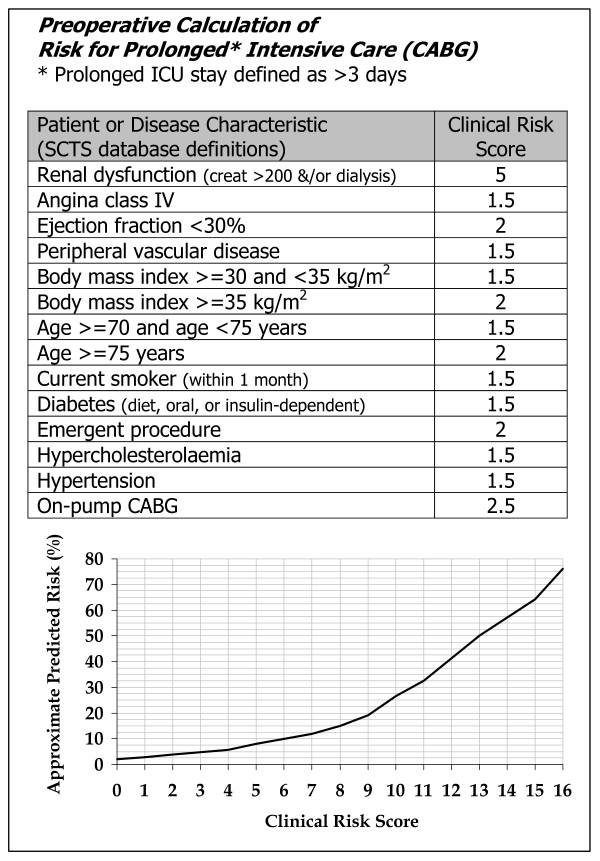
Simplified clinical risk assessment tool for prolonged intensive care unit stay.

**Figure 5:**
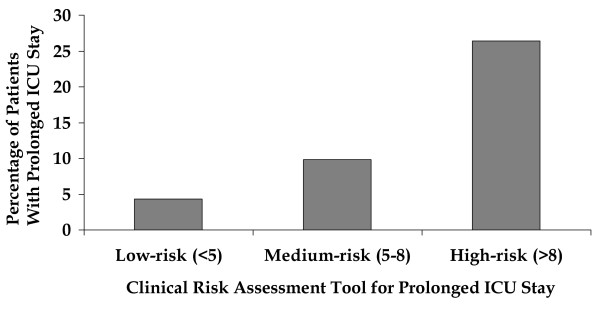
Comparison of off-pump and on-pump coronary surgery by prolonged intensive care unit stay risk groups.

A simplified clinical risk assessment tool derived from the logistic regression equation, described at the bottom of Table 2, is shown in Figure 4. The clinical risk assessment tool was split into low, medium, and high risk groups as shown in Figure 5.

## 4. Discussion

In this study, we developed and validated a multivariate preoperative risk prediction model for the risk of prolonged ICU stay. The prediction model uses 12 readily available preoperative patient and disease characteristics assigning an independent weight to each to provide quantitative information about risk. The prediction model demonstrated relatively strong discriminatory ability (area under ROC curve = 0.72), with no significant deviation from perfect fit. This prediction model, which makes use of routinely available preoperative data, can serve the clinician and the patient by providing a simple method to assess accurately the risk of prolonged ICU stay following CABG surgery. Also, by accounting for patient variability, the model may provide an estimate for required resources in CABG surgery and help efforts to control costs and avoid bed shortages in ICU.

Because of the ever-present shortage of beds within hospitals, especially within ICU, considerable effort needs to be expended in resource planning and allocation. One solution to increase efficiency in the field of cardiac surgical ICUs is to plan the operations to use available resources in an optimal fashion [12]. Prediction of postoperative ICU stay would facilitate decisions to allocate resources and to plan weekly schedules for CABG operations. When ICU bed availability is an issue, patients with high risk of staying for a prolonged stay could be electively scheduled for surgery in a series rather than parallel. While scheduling the operative list, a case mix of patients that includes patients with probability of prolonged ICU stay and those who are likely to have uncomplicated recovery could potentially avoid the possibility of blocking beds in ICU.

Existing risk stratification models for mortality following CABG have been shown to be good predictors of prolonged ICU stay. Lawrence and co-workers concluded that the Parsonnet score was a good predictor of ICU stay <24 hours, which could help cardiothoracic units when resources are limited to a few ICU beds [13]. Nilsson and associates also found the EuroSCORE to be a useful predictor of ICU stays greater than two days in open heart surgery [14]. However, our study found that, compared to a specifically designed prediction model for prolonged ICU stay, the Parsonnet, and both versions of the EuroSCORE were not reliable predictors, with a tendency to under-predict.

Several studies have identified risk factors for prolonged ICU stay with varying definitions. Wong and colleagues examined 885 CABG patients and defined prolonged ICU stay as greater than 48 hours. Also, unlike our study, they examined post-operative factors. The risk factors identified included increased age, female sex, pre-operative myocardial infarction, post-operative use of intra-aortic balloon pump, inotropes, bleeding, atrial arrhythmia and renal insufficiency [2]. Michalopoulos and co-workers used the same definition as Wong, and included perioperative factors such as blood use and inotrope support in their final logistic regression model, with only age and ejection fraction identified as preoperative risk factors [3]. Other postoperative factors identified as predictors of prolonged ICU stay have included elevated Troponin- T levels [15] and pulmonary artery blood temperature greater than 36.4 degrees C on admission to the ICU [16]. Inclusion of peri- or post-operative factors in our study, however, would have limited the usefulness of the prediction model in aiding resource management prior to surgery.

Christakis and colleagues [4] analysed preoperative risk factors for prolonged ICU stay in 889 consecutive patients undergoing isolated CABG between 1990 and 1992. Using the same definition as in our study, 6.8% of patients had stays in ICU of greater than 3 days. Only two preoperative risk factors however could be identified, with both recent myocardial infarction and current smoking increasing the risk of prolonged ICU stay. Our study also found an association between smoking and prolonged intensive care, however, a history of, or recent, myocardial infarction was not identified as a risk factor.

More recently, Janssen and associates [5] published a preoperative prediction model for prolonged ICU stay defined as greater than 3 days. The analysis included 888 contemporary CABG patients, of which 104 stayed in ICU for more than 3 days. They presented a logistic regression equation to predict prolonged ICU stay, which included the following variables: lung disease, no-sinus rhythm, no mild valve pathology, prior surgery, priority, and on-pump surgery. These factors are quite different from those identified in our study, except for priority and on-pump surgery. A reason for this might be due to the sample size, with only 104 outcomes compared to the 457 prolonged ICU stay patients in our analysis. Inclusion of on-pump surgery as a preoperative factor is based on the fact that in most circumstances, the use of CPB is a preoperative planned approach and not necessarily an intra-operative decision, except in a minority of cases. There may be some concern that on-pump surgery would be identified as a risk factor purely due to selection bias with off-pump surgery being performed in lower-risk cases. However, Bucerius and colleagues concluded, in an analysis which included over 700 off-pump CABG, that avoiding cardiopulmonary bypass could optimize patient outcome with respect to prolonged ICU stay [17].

There are limitations to this study which need to be considered. One such limitation is that it is based on data from one institution, and therefore, subject to the efforts of local practices and case mix. Although we have validated the prediction model on external data between April 2003 and March 2004, this model still requires validation from other institutions. It is also unclear how useful this tool might actually be in aiding resource management, compared to a clinicians own estimates of risk for an individual patient. It has been shown though that clinicians tend to overestimate the probability of mortality and prolonged ICU stay [18]. The application of this model to other cardiac surgery procedures is also needed as these other non-CABG procedures could have a significant impact on ICU bed availability.

In conclusion, clinicians may use the prediction model contained within this paper to aid in resource management within the ICU. The logistic version of the model can be easily programmed into appropriate software resident on desktops and hand-held computers. Alternatively, the clinical risk assessment tool could be used and provided on small pocket-sized laminated cards providing simple and easy approximations of the risk of prolonged ICU.
